# Clinical Characteristics in Panic Disorder Patients in Emergency Department

**DOI:** 10.1192/j.eurpsy.2023.1428

**Published:** 2023-07-19

**Authors:** B. Nam

**Affiliations:** Psychiatry, Dr. Nam’s Psychiatric Clinic, Chungju, Korea, Republic Of

## Abstract

**Introduction:**

Panic disorder is a widespread mental illness associated with the use of high levels of emergency care. However, the clinical characteristics of patients with panic disorder in the emergency department are not known.

**Objectives:**

This study was designed to investigate datas related to panic attack and treatment in emergency room of panic disorder patients who visited emergency room for panic attack.

**Methods:**

A retrospective analysis of medical records was conducted on 92 patients with panic disorder who visited Chungju Konkuk university hospital emergency department due to panic attack and had bodily symptoms from 1st January 2010 to 31th December 2019. In addition to demographic characteristics and comorbid disorders, triggering stressors and alcohol consumption were corrected as pre-panic attack datas, bodily symptoms at the time of panic attack were corrected as datas during attack, electrocardiogram trial, consultation with psychiatrist, admission and information of used psychotropic drugs were corrected as post-attack data. Depending on size of data, Chi-square test or Fisher’s exact test was used. Collected data was analyzed using R 4.03.

**Results:**

Cardiovascular disease was accompanied by 5.4% and depressive disorder was the most common coexisting mental disorder. Among triggering stressors, economic problem/work-related stress was significantly higher in men than women (x2=4.322, p<0.005). The most common physical symptom during attack was circulatory (65.2%), followed by respiratory (57.6%), numbness-paralysis (33.7%), dizziness (19.6%), gastro-intestinal (14.1%) and autonomic symptom (12.0%). Electrocardiogram was taken at higher rate when patients complained circulatory symptom (x2=8.46, p<0.005). The psychotropic drug most commonly used in emergency room was lorazepam, used in 92.1%.

**Conclusions:**

The most common bodily symptom during panic attack was circulatory symptom and the most common triggering stressor in men was economic problem/work-related stress. The most commonly used psychotropic for panic attack was lorazepam.

**Disclosure of Interest:**

None Declared

